# COVID-19 and social distancing among children and adolescents in Brazil

**DOI:** 10.11606/s1518-8787.2021055003832

**Published:** 2021-06-18

**Authors:** Fernando C Barros, Fernando P Hartwig, Aluísio J D Barros, Ana M B Menezes, Bernardo L Horta, Cláudio J Struchiner, Luis Paulo Vidaletti, Mariangela F Silveira, Marilia A Mesenburg, Odir A Delagostin, Pedro C Hallal, Cesar G Victora

**Affiliations:** I Universidade Católica de Pelotas PelotasRS Brasil Universidade Católica de Pelotas. Curso de Pós-Graduação em Saúde no Ciclo Vital. Pelotas, RS, Brasil; II Universidade Federal de Pelotas Faculdade de Medicina Departamento de Medicina Social PelotasRS Brasil Universidade Federal de Pelotas. Faculdade de Medicina. Departamento de Medicina Social. Pelotas, RS, Brasil; III Fundação Getúlio Vargas Escola de Matemática Aplicada Rio de JaneiroRJ Brasil Fundação Getúlio Vargas. Escola de Matemática Aplicada. Rio de Janeiro, RJ, Brasil; IV Universidade Federal de Ciências da Saúde de Porto Alegre Faculdade de Medicina Porto AlegreRS Brasil Universidade Federal de Ciências da Saúde de Porto Alegre. Faculdade de Medicina. Porto Alegre, RS, Brasil

**Keywords:** Coronavirus Infections, prevention & control, Child, Adolescent, Social Isolation, Socioeconomic Factors

## Abstract

**OBJECTIVE:**

To estimate the prevalence of SARS-CoV-2 antibodies and the adherence to measures of social distancing in children and adolescents studied in three national surveys conducted in Brazil between May–June 2020.

**METHODS:**

Three national serological surveys were conducted in 133 sentinel cities located in all 27 Federative Units. Multistage probability sampling was used to select 250 individuals per city. The total sample size in age ranges 0–9 and 10–19 years old are of 4,263 and 8,024 individuals, respectively. Information on children or adolescents was gathered with a data collection app, and a rapid point-of-case test for SARS-CoV-2 was conducted on a finger prick blood sample.

**RESULTS:**

The adjusted prevalence of antibodies was 2.9% (2.2–3.6) among children 0–9 years, 2.2% (1.8–2.6) among adolescents 10-19 years, and 3.0% (2.7–3.3) among adults 20+years. Prevalence of antibodies was higher among poor children and adolescents compared to those of rich families. Adherence to social distancing measures was seen in 72.4% (71.9–73.8) of families with children, 60.8% (59.6–61.9) for adolescents, and 57.4% (56.9–57.8) for adults. For not leaving the house except for essential matters the proportions were 81.7% (80.5–82.9), 70.6% (69.6–61.9), and 65.1% (64.7–65.5), respectively. Among children and adolescents, social distancing was strongly associated with socioeconomic status, being much higher in the better-off families.

**CONCLUSIONS:**

The prevalence of antibodies against SARS-CoV-2 showed comparable levels among children, adolescents, and adults. Adherence to social distancing measures was more prevalent in children, followed by adolescents. There were important socioeconomic differences in the adherence to social distancing among children and adolescents.

## INTRODUCTION

The pandemic of Covid-19 seems to affect children less often and with lower severity than adults^1^. In the United States, the CDC reported 2,572 (1.8%) infections < 18 years of age in 150,000 cases^2^. In China, only 1% were between 0–9 years old and 1% 10–19 years old in 44,000 cases^3^.

In 47 studies, no differences in prevalence were observed between children/adolescents and other age groups^4^. However, in a review of 10 studies, with three national surveys, the prevalence among children between 0–9 years old was lower than for adults; in most publications, adolescents and adults showed similar prevalence^5^. In Spain, prevalence was 2.5% among children < 5 years old, 4.7% for 5–9 years old, 5.5% for adolescents, and 6.6% over 19 years of old^6^; in Iceland, prevalence among children < 10 years old was 6.7%, and 13.7% over 10 years old^7^.

In 32 studies, children and adolescents were less susceptible to the SARS-CoV-2 virus, with a 0.56 (0.37–0.85) odds ratio for becoming an infected contact, compared to adults^5^. In another 18 papers of SARS-CoV-2 infections, most cases presented mild respiratory symptoms or were asymptomatic, with one case of severe infection, and no deaths were reported in children between 0–9 years^8^. There are, however, reports of cases of severe acute Covid-19 and multisystem inflammatory syndrome in children (MIS-C)^9^.

Several reasons are proposed to explain why SARS-CoV-2 infections are less severe in children^10^. Cross reactivity to previous coronavirus infections is more frequent among children^11^; a significant inverse correlation between mumps antibody titers provided by the MMR II vaccine and the severity of Covid-19 symptoms was reported^12^.

It is not known if measures of social distancing are equally followed by different age groups, it is possible that the lower prevalence of Covid-19 among children could be partially explained by spending less time in outdoor activities and having lower exposure to contaminated people^13^.Furthermore, families with children might be more adherent to measures of social distancing; in a Cyprus study, 72% of children complied with most of the non-pharmacological interventions^14^.

Little is known about how adolescents adopt social distancing; studies have shown that they do so because of a sense of social responsibility to prevent others from becomingsick^15,16^.

This paper estimates the prevalence of antibodies against SARS-CoV-2, and compliance with measures of social distancing among children and adolescents, as compared to adults, using three nationwide community surveys conducted in Brazil. To our knowledge, this is the largest study available on these issues.

Over 30% of the world’s population are under 18 years (2.3 billion people)^17^. Population under 18 years are nearly 50% in Sub-Saharan Africa and 18.6% in Western Europe; in Brazil, it is 26.1%^17^. All these children and adolescents are not going to be immunized against the SARS-CoV-2 virus at least in the next months, since the vaccines currently in use do not include <18-year-old subjects in their target population. This is the reason why studying the prevalence of Covid-19 infections in children and adolescents and the patterns of their behavior regarding measures of social distancing is particularly important.

## METHODS

Brazil’s 26 States and the Federal District are divided into 133 intermediate regions; the largest city in each region constitutes a sentinel city. Three nationwide serological survey were conducted in May 14–21, June 4–7, and June 21–23 in these cities. An account of the methodology is available elsewhere^18,19^.

In each survey, multistage sampling was used to select 25 urban census tracts in each city and 10 households within each tract, totaling 250 individuals per city and 33,250 individuals for each survey. A data collection app selected one individual among all household members; this person was replaced by a second member if the first refused to provide a blood sample.

In the first survey, the field workers were able to test 25,025 individuals(75.3% of the desired sample). During the home visits, a rapid point-of-care test was performed, the WONDFO SARS-CoV-125 2 Antibody Test (Wondfo Biotech Co., Guangzhou, China), using finger prick blood samples in the selected subject, and a short questionnaire was applied including sociodemographic information and compliance with social distancing measures.

In the second survey round, the same sampling scheme was used, with different household being randomly chosen in the same census tracts as in the first round. The field team was able to interview and test 31,165 subjects (93.7%). The third round used the same sampling scheme, and 33,205 subjects (99.8%) were interviewed and tested. Children under the age of 1 year were included only in the third survey, since it was assumed that parents would not allow a fingerprick sample to be obtained from infants.

Field workers used tablets or smartphones to record the full interviews, register all answers, and photograph test results.

Ethnic group affiliation was assessed with using the official Brazilian classification with five groups, based on the question: “What is your race or color?” The response options are “white”, “brown”,“black”, “yellow (Asian)” and “indigenous”. This system is endorsed by the National Institute of Geography and Statistics and by minority groups.

A household wealth index was created based on principal component analysis of a list of assets and goods (computer or laptop, internet access, color television, air conditioning equipment, number of vehicles, cable TV, number of bathrooms and number of bedrooms)^20^. The first component was extracted and then divided into quintiles; the first quintile represents the 20% poorest households, the fifth quintile represents the wealthiest 20%.

Social distancing measures were evaluated with two questions. The first one was “To what extent are you managing to follow the social distancing guidance set by the health authorities, i.e., staying at home and avoiding contact with others?”. The answers were recorded in two groups: those following more strictly the recommendations, staying at home all the time or nearly all the time (alternatives d and e) and the remainder of the sample.

The second question was, “What have your routine activities been like?”. This question categorized in two groups: those staying at home all the time or leaving home only for essentials, and the remainder.

Finally, respondents were asked if they used face masks when leaving home. This question had direct answers “yes” or “no.”

If the randomly selected respondent was a child (under age 12) or an older adult that was unable to answer, the question was asked to the respondent’s legal guardian.

The point of care rapid test underwent four independent validation studies and pooling the results, weighted by sample sizes, sensitivity was estimated at 84.8% (95%CI 81.4%–87.8%) and specificity at 99.0% (95%CI 97.8%–99.7%)^21^. We conducted a household survey in the state of Rio Grande do Sul^22^. Of 4,188 participants, we found only two (< 1%) positive results. Assuming that all cases in that survey were false positives leads to a specificity of 99.95%.

From mid-October to mid-November 2020, we conducted another validation study to assess the possibility that sensitivity changes over time. The sensitivity ranged from ~40% (among subjects tested five months after the positive RT-PCR result) to ~80% (among subjects tested within two months)^23^. Therefore, the correction parameters used in the present analyses were: 99·95% as the test specificity; and a sensitivity value estimated using the strategy described below to account for variation over time.

To account for the time-dependent decline in sensitivity, we estimated the time distribution of the pandemic in each of the 133 cities. We used official statistics of Covid-19 death rates, which are more reliable than reported cases, which, in turn, depend on intensity of testing. However, using mortality data for this purpose requires assuming that disease fatality is similar between cities and over time, so that differences in death rates reflect differences in infection rates. We also assumed that infected individuals that died had become positive in the RT-PCR two weeks before dying. For each phase of the survey, the sensitivity value used to correct the unadjusted prevalence represented a weighted average of the sensitivity values obtained in our second validation study^22^, with the Covid-19 mortality rate curve and the number of positive tests in the study in each city providing the statistical weights. Weighting by the death curve allows incorporating the time-dependent decay in the calculation. For example, if most deaths in a city were recent, sensitivity had a higher value than in another city where most deaths had occurred in the past. Weighting by the number of positive tests results in the death curve in cities with more individuals that tested positive having a greater weight than cities with less individuals that tested positive. Taking to the extreme, had all positive cases occurred in a single city, then only the death curve of this city would be used to estimate the sensitivity, which is a desired property. Uncertainty in the model that describes the relationship between sensitivity and time was incorporated by re-sampling the model coefficients in the parametric bootstrap procedure implemented in the previously developed correction method.

In analyses combining the three phases, results were generated for each phase separately and then combined using weighted fixed effects meta-analysis, in which the weights correspond to sample size. In stratified analyses, the number of positive test results in each city used for weighting were stratum specific. However, the death curves in each city were assumed to be the same as the overall curves due to lack of official stratum-specific data for all stratifiers we considered^18^.

All analyses were performed using R (version 4.0.2). The “survey”^24,25^ and “metafor”^26^ packages were used to account for the sampling design of the survey and for meta-analysis, respectively.

All interviewers were tested and found to be negative for the virus before field work and were provided with individual protection equipment discarded after each visit. Ethical approval was obtained from the Brazilian’s National Ethics Committee (process number CAAE 193 30721520.7.1001.5313), with written informed consent from all participants. Positive cases were reported to the municipal Covid-19 surveillance systems. Data is publicly available upon request from the authors.

## RESULTS

In the three surveys, 89,362 individuals were interviewed and tested, of whom 2,064 (2.3%) tested positive for SARS-CoV-2. These include 4,263 children (aged 0–9 years old), 8,024 adolescents (aged 10–19 years old), and 77,075 adults (aged 20+ years old).

Table 1 shows the prevalence of positive antibody tests – unadjusted and adjusted for sample design and test validity – for each age group for the pooled data. The adjusted prevalence was 2.9% (2.2–3.6) for children, 2.2% (1.8–2.6) for adolescents, and 3.0% (2.7–3.3) for adults. In all age groups, antibody prevalence was highest in the North (Amazon) Region – 6.8% for children – followed by the Northeastern Region; in the remaining regions, seroprevalence was 1% or below. There were marked socioeconomic differences in the prevalence of SARS-CoV-2 antibodies within each age group: for children, the poorest socioeconomic quintile presented a prevalence three times higher than the richest quintile, and for adolescents this ratio was equal to two.

**Table 1:** Seroprevalence according to sociodemographic characteristics, by age group.

	Sample distribution		Unadjusted		Adjusted for sample design and test validity	
	Number	%	Estimate	95%CI	Estimate	95%CI
Age 0–9						
Region			0.001		0.001	
North	1,041	24.4%	5.4%	4.1–6.4	6.8%	4.9–8.8
Northeast	1,222	28.7%	2.5%	1.7–3.4	3.1%	1.9–4.3
Central-West	512	12.0%	0.8%	0.3–2.0	1.0%	0.0–2.0
Southeast	872	20.5%	0.7%	0.3–1.4	0.8%	0.1–1.5
South	617	14.5%	0.3%	0.1–1.1	0.4%	0.0–1.1
Gender			0.491		0.873	
Female	2,028	46.9%	2.3%	1.7–3.0	2.8%	2.0–3.7
Male	2,235	53.1%	2.3%	1.7–3.0	2.9%	2.1–3.8
Wealth quintiles			0.045		0.054	
Poorest	1,195	28.0%	3.1%	2.2–4.2	3.9%	2.6–5.3
2nd	983	20.9%	2.1%	1.3–3.3	2.7%	1.4–4.0
3rd	903	21.2%	1.8%	1.0–2.8	2.2%	1.1–3.4
4th	683	16.0%	2.8%	1.7–4.3	3.5%	1.9–5.2
Richest	589	13.8%	1.2%	0.5–2.4	1.4%	0.3–2.6
Ethnicity			0.001		0.001	
White	1,528	36.3%	1.0%	0.6–1.6	1.3%	0.6–2.0
Black	329	7.8%	1.8%	0.8–3.9	3.8%	2.8–4.8
Brown	2,209	52.4%	3.0%	2.3–3.7	2.3%	0.3–4.3
Asian	97	2.3%	3.1%	1.0–8.7	4.0%	0.0–8.8
Indigenous	49	1.2%	10.2%	4.4–21.7	13.3%	2.2–24.4
Household size			0.091		0.240	
1	0	0.0%	--	--		
2	187	4.4%	1.6%	0.5–4.6	2.0%	0.0–4.3
3	996	23.4%	2.1%	1.3–3.2	2.7%	1.5–3.9
4	1,396	32.7%	1.8%	1.2–2.6	2.2%	1.3–3.2
5+	1,684	39.5%	2.9%	2.2–3.8	3.7%	2.6–4.8
All	4,263	100.0%	2.3%	1.8–2.7	2.9%	2.2–3.6
Age 10–19						
Region			0.001		0.001	
North	1,897	23.6%	4.5%	3.6–5.5	5.6%	4.3–6.9
Northeast	2,554	31.8%	2.0%	1.4–2.5	2.5%	1.7–3.2
Central-West	785	9.8%	0.3%	0.1–0.9	0.3%	0.0–0.8
Southeast	1,629	20.3%	0.2%	0.1–0.6	0.3%	0.0–0.6
South	1,159	14.4%	0.1%	0.02–0.4	0.1%	0.0–0.4
Gender			0.284		0.546	
Female	4,189	54.9%	1.9%	1.4–2.3	2.3%	1.7–2.9
Male	3,835	45.1%	1.7%	1.3–2.1	2.1%	1.5–2.6
Wealth quintiles			0.004		0.023	
Poorest	1,675	20.9%	2.1%	1.5–2.8	2.6%	1.7–3.5
2nd	1,506	18.8%	2.5%	1.7–3.3	3.1%	2.0–4.2
3rd	1,674	20.9%	1.7%	1.2–3.4	2.2%	1.3–3.0
4th	1,670	20.8%	1.6%	1.0–2.2	1.9%	1.1–2.7
Richest	1,498	18.7%	1.0%	0.6–1.6	1.2%	0.5–1.9
Ethnicity			0.001		0.001	
White	2,468	31.2%	1.0%	0.6–1.4	1.2%	0.7–1.7
Black	945	12.0%	2.1%	1.0–2.7	2.6%	2.0–3.2
Brown	4,147	52.5%	1.7%	1.6–2.5	2.1%	1.0–3.2
Asian	217	2.7%	1.4%	0.4–3.9	1.8%	0.0–4.2
Indigenous	124	1.6%	7.3%	3.8–13.2	9.2%	3.3–15.2
Household size			0.301		0.072	
1	298	3.7%	0.3%	0.1–1.8	0.4%	0.0–1.6
2	1,087	13.5%	1.8%	1.1–2.8	2.3%	1.2–3.4
3	1,946	24.3%	1.7%	1.2–2.3	2.1%	1.3–2.9
4	2,279	28.4%	1.9%	1.4–2.5	2.4%	1.6–3.2
5+	2,414	30.1%	1.8%	1.3–2.4	2.3%	1.5–3.0
All	8,024	100%	1.8%	1.5–2.1	2.2%	1.8–2.6
Age 20+						
Region			0.0001		0.001	
North	13,075	17.0%	7.1%	6.6–7.5	8.9%	7.9–9.9
Northeast	23,033	29.9%	3.0%	2.8–3.2	3.8%	3.4–4.3
Central-West	8,496	11.0%	0.4%	0.3–0.6	0.5%	
Southeast	19,359	25.1%	0.7%	0.6–0.8	0.9%	0.7–1.1
South	13,112	17.0%	0.2%	0.1–0.3	0.2%	0.1–0.3
Gender			0.390		0.843	
Female	45,836	59.5%	2.4%	2.2–2.5	3.0%	2.7–3.3
Male	31,239	40.5%	2.3%	2.2–2.5	2.9%	2.6–3.3
Wealth quintiles			0.001		0.001	
Poorest	18,106	23.5%	3.0%	2.7–3.2	3.8%	3.3–4.3
2nd	14,161	18.4%	2.8%	2.5–3.0	3.5%	3.0–4.0
3rd	14,748	19.1%	2.3%	2.1–2.5	2.9%	2.5–3.3
4th	14,909	19.3%	2.2%	1.9–2.4	2.7%	2.3–3.1
Richest	15,143	19.6%	1.4%	1.2–1.6	1.7%	1.5–2.0
Ethnicity			0.001		0.001	
White	28,387	37.7%	1.2%	1.0–1.3	1.4%	1.2–1.7
Black	10,030	13.3%	2.6%	2.3–2.9	4.1%	3.6–4.5
Brown	33,732	44.8%	3.2%	3.0–3.4	3.3%	2.8–3.8
Asian	2,132	2.6%	2.2%	1.6–2.8	2.7%	1.9–3.6
Indigenous	1,046	1.4%	5.0%	3.8–6.4	6.3%	4.5–8.1
Household size					0.001	
1	17,084	22.2%	2.2%	1.9–2.4	2.7%	2.3–3.1
2	25,221	32.7%	2.1%	1.9–2.2	2.6%	2.3–2.9
3	15,432	21.3%	2.2%	1.9–2.4	2.8%	2.4–3.2
4	10,339	13.4%	2.5%	2.2–2.8	3.2%	2.7–3.6
5+	7,999	10.4%	3.9%	3.4–4.3	4.9%	4.2–5.6
All	77,075	100.0%	2.4%	2.2–2.5	3.0%	2.7–3.3

Regarding ethnicity, indigenous children and adolescents represented less than 2% of the sample and presented markedly higher antibody prevalence that the other ethnic groups, reaching 13.3% and 9.2%, respectively. White children and adolescents showed prevalence a little higher than 1.0%, whereas the other three groups – brown, blacks and Asians –, showed intermediate levels. The same pattern was observed, though a little less marked, amongst adults. Regarding prevalence by the number of people in the household, associations within children and adolescents were not significant, whereas the prevalence increased in adults with household size.

Table 2 presents the prevalence of reported compliance with social distancing and face mask use, by age groups. Children, followed by adolescents, were more likely to comply with social distancing recommendations than adults; adherence to distancing measures was seen in 72.4% (71.9–73.8) of families with children as sampled subjects, 60.8% (59.6–61.9) for adolescents, and 57.4% (56.9–57.8) for adults. For not leaving the house except for essential matters the proportions were 81.7% (80.5–82.9), 70.6% (69.6–61.9), and 65.1% (64.7–65.5), respectively. Reported use of face masks, on the contrary, was nearly universal among adolescents and adults, and less common among children – 89.8% (88.8–90.7). Prevalence of face mask use was 78.9% (76.8–80.8) among0–4-year olds and 96.8% (96.0–97.4) among 5–9-year olds (not shown in the Table).

**Table 2:** Prevalence of subjects that followed measures of social distancing, who stayed at home most of the time and used face mask by age group.

Individual-level behaviors (%)			
Age	Compliance with social distancing measures	Staying at home most of the time	Use of face mask
	p < 0.001	p < 0.001	p < 0.001
0–9	72.4 (71.0–73.8)	81.7 (80.5–82.9)	89.8 (88.8–90.7)
10–19	60.8 (59.6–61.9)	70.6 (69.6–71.6)	96.6 (96.2–97.0)
20+	57.4 (56.9–57.8)	65.1 (64.7–65.5)	98.6 (98.5–98.7)

**Figure 1:**
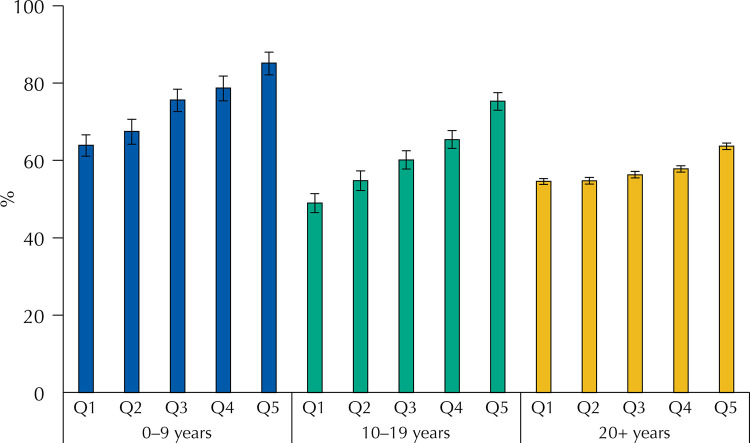
Prevalence of subjects that followed measures of social distancing stratified by age and socioeconomic status.

**Figure 2:**
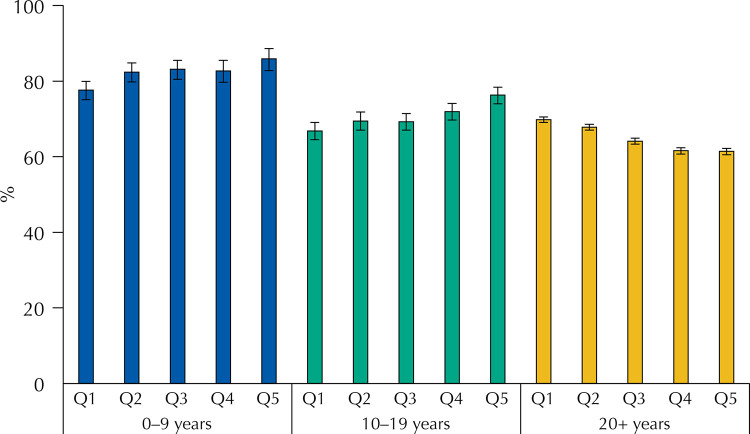
Prevalence of subjects that stayed at home or left home only for essential activities stratified by age and socioeconomic status.

**Figure 3:**
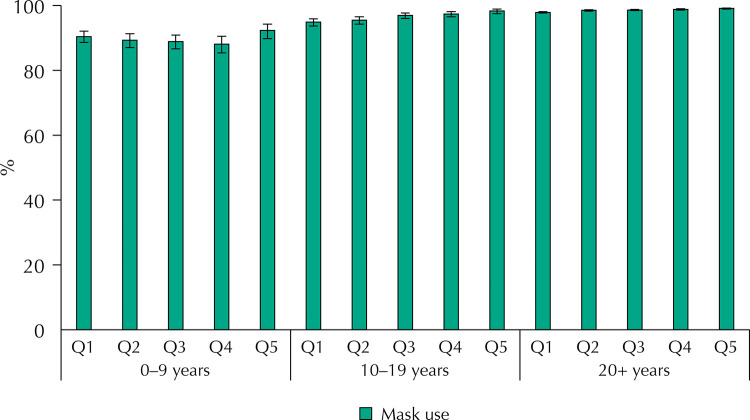
Prevalence of use of face mask when leaving the house stratified by age and socioeconomic status.

Figures 1, 2, and 3 present the distribution of compliance with social distancing recommendations and with staying at home and use of face mask when leaving home by age groups, stratified by quintiles of socioeconomic position. For the three safety measure outcomes, there was strong statistical evidence (p < 0.001) for interaction between age and socioeconomic position. For children and adolescents in the wealthiest quintile, compliance with recommendations (Figure 1) was more than 20 percent points higher than in the poorest quintile – 85.2%, compared to 63.9% (p for linear trend < 0.001) for children and more than 25 percent points for adolescents – 75.3%,compared to49.0% (p for linear trend < 0.001). Adults also presented a less marked trend in the same direction (p < 0.001). Compliance with remaining at home (Figure 2) increased with socioeconomic position for children (p < 0.001) and adolescents (p < 0.001), but the opposite trend was observed among adults, with a difference between the richest and poorest quintiles of 8.4 percent points (p < 0.001). Regarding the use of face masks, the prevalence was high in all socioeconomic groups, but among adolescents and adults there was a significant trend towards more use in the wealthiest quintiles (p < 0.001), whereas no significant differences were observed for children.

## DISCUSSION

In our paper, we discuss the findings of SARS-CoV-2 antibody seroprevalence and social distancing measures of more than 4,000 children and 8,000 adolescents that were studied in three national surveys in 133 sentinel cities in Brazil between May 14 and June 23, 2020. Although not considered a representative sample of the whole country, since rural areas and small towns were not included, the survey displayed a picture of the largest cities of the country located in all 27 States and the Federal District. Some of the results of these surveys have been already published^18,27^.

Compared with the overall distribution of the Brazilian population, our sample had more individuals from the North and Northeastern regions, and fewer children and adolescents. In our sample, 4.8% were in the 0–9 years groups, compared to 13.5% in the country population, for adolescents between 10–19 yearsold, our proportion was 9.0% whereas the country distribution is 15.4%. It is possible that children were under-represented because they, and their parents, were reluctant in undergoing a finger prick to collect a blood sample for the serological test. Furthermore, children under 12 months were excluded in the first two surveys.

This lower, however expected, proportion of children and adolescents is a limitation of our study, together with the use of sentinel cities that do not represent the whole Brazilian population, leaving aside small cities and rural population (15% of the country’s population). One strength of the study was its population-based multistage sampling design, since most large sample studies rely upon conveniency samples often collected in health facilities.

Unlike some other studies^5^thatshowed lower antibody prevalence among children, in our population, children and adolescents presented seroprevalence comparable to those observed in adults. We are not able to determine ifthe bias introduced by restrictions in the number of children in our sample played a role in these results.

Socioeconomic and ethnic inequalities were clearly observed, with the prevalence of SARS-CoV-2 antibodies being two to three times higher amongst children and adolescents of families in the lowest socioeconomic quintile compared to those in the highest quintile. Moreover, prevalence amongst the small group of indigenous children and adolescents was 7 to 10 times higher than that amongst white; Asian, Brown, and Black children were also more likely to present antibodies compared to white children. We had already reported on the high prevalence of antibodies in indigenous populations in our previous publications^18,27^, in which we show that this is mostly due to geographic reasons, as most of these indigenous children and adolescents live in the North Region, where the prevalence of infection was the highest in the country at the time of the study. To our knowledge, this is the first study reporting socioeconomic and ethnic differences in seroprevalence among children.

We found that families of which the sampled subject was a child were much more adherent to the distancing measures indicated by the health authorities, followed by families of adolescents, in comparison of families where the sampled subject was an adult. Families with children as the sampled subject had a prevalence of staying most of the time at home leaving only for essential needs which was 10 percent points higher than that of adolescents’ families, and 20 percent points higher than those of adults.

Although it is argued that one possible explanation for lower Covid-19 infection rates among children is because they have fewer outdoor activities and are less likely to travel by air^13^, we could not find any reports in the literature comparing levels of social distancing among children, adolescents, and adults.

The finding that compliance with social distancing among children and adolescents was inversely associated with their families’ socioeconomic position is particularly important. Schools have been closed since March 2020, so that children were protected from exposure. It is generally recognized that poor families in low and middle-income countries have greater difficulty in implementing effective preventive measures such as social distancing and personal hygiene^28^. Multiple reasons are possibly involved in these large differences in the level of compliance with social distancing, such as lower literacy and less awareness about the need for protection, overcrowded households where parents are unable to keep their children inside most of the time, and weaker family safety nets that may force parents to take their children along when they leave home for work, particularly in the informal sector.The lower observance of distancing measures among poorer families could be one important reason for the higher prevalence of SARS-CoV-2 antibodies observed among poor children and those of disadvantaged ethnic minorities when compared with those from families in better socioeconomic position.

The prevalence of antibodies against SARS-CoV-2 in Brazil was similar in children, adolescents, and adults, being two to three times higher among the poorest children and adolescents when compared with the richest. Adherence to measures of social distancing was higher in families with children, followed by those with adolescents, and, finally, those with adults. Among children and adolescents, there were important differences in the proportions regarding following the distancing recommendations, with more privileged families being much stricter than those poorest. Such inequalities in social distancing might be one of the reasons behind socioeconomic and ethnic differences in the prevalence of antibodies to SARS-CoV-2. Moreover, since Covid-19 vaccines currently used in several countries are not yet recommended for children and adolescents up to 18 years old, measures of social distancing will continue to be particularly important in the near future for young age groups. Finally, future studies should evaluate if the progressive levels of immunization of adults, resulting from vaccination campaigns, will change their adherence to social distancing measures that also protect their children and adolescents.
